# The global prevalence of gastric cancer in *Helicobacter pylori*-infected individuals: a systematic review and meta-analysis

**DOI:** 10.1186/s12879-023-08504-5

**Published:** 2023-08-19

**Authors:** Maryam Shirani, Reza Pakzad, Mohammad Hossein Haddadi, Sousan Akrami, Arezoo Asadi, Hossein Kazemian, Melika Moradi, Vahab Hassan Kaviar, Abolfazl Rafati Zomorodi, Saeed Khoshnood, Mahnaz Shafieian, Ronia Tavasolian, Mohsen Heidary, Morteza Saki

**Affiliations:** 1 Toxicology Research Center, Medical Basic Sciences Research Institute, Ahvaz Jundishapur University of Medical Sciences, Ahvaz, Iran; 2 Department of Epidemiology, Faculty of Health, Ilam University Medical Sciences, Ilam, Iran; 3 Student Research Committee, Ilam University of Medical Sciences, Ilam, Iran; 4 Clinical Microbiology Research Center, Ilam University of Medical Sciences, Ilam, Iran; 5 Students’ Scientific Research Center (SSRC), Tehran University of Medical Sciences, Tehran, Iran; 6 Department of Microbiology, Faculty of Medicine, Ahvaz Jundishapur University of Medical Sciences, Ahvaz, Iran; 7 Endocrine Research Center, Institute of Endocrinology and Metabolism, Iran University of Medical Sciences, Tehran, Iran; 8 Department of Medical Microbiology, Faculty of Medicine, Ilam University of Medical Science, Ilam, Iran; 9 Department of Bacteriology and Virology, School of Medicine, Shiraz University of Medical Sciences, Shiraz, Iran; 10 Department of Midwifery, Faculty of Nursing and Midwifery, Ilam University of Medical Sciences, Ilam, Iran; 11 Department of Medicine, Faculty of Nutrition Science, University of Cheste, Chester, UK; 12 Department of Laboratory Sciences, School of Paramedical Sciences, Sabzevar University of Medical Sciences, Sabzevar, Iran; 13 Cellular and Molecular Research Center, Sabzevar University of Medical Sciences, Sabzevar, Iran

**Keywords:** Infection, Prevalence, Gastric cancer, *Helicobacter pylori*, Systematic review, Meta-analysis

## Abstract

**Background:**

*Helicobacter pylori* is a gastrointestinal pathogen that infects around half of the world's population. *H. pylori* infection is the most severe known risk factor for gastric cancer (GC), which is the second highest cause of cancer-related deaths globally. We conducted a systematic review and meta-analysis to assess the global prevalence of GC in *H. pylori*-infected individuals.

**Methods:**

We performed a systematic search of the PubMed, Web of Science, and Embase databases for studies of the prevalence of GC in *H. pylori*-infected individuals published from 1 January 2011 to 20 April 2021. Metaprop package were used to calculate the pooled prevalence with 95% confidence interval. Random-effects model was applied to estimate the pooled prevalence. We also quantified it with the I^2^ index. Based on the Higgins classification approach, I^2^ values above 0.7 were determined as high heterogeneity.

**Results:**

Among 17,438 reports screened, we assessed 1053 full-text articles for eligibility; 149 were included in the final analysis, comprising data from 32 countries. The highest and lowest prevalence was observed in America (pooled prevalence: 18.06%; 95% CI: 16.48 − 19.63; I^2^: 98.84%) and Africa (pooled prevalence: 9.52%; 95% CI: 5.92 − 13.12; I^2^: 88.39%). Among individual countries, Japan had the highest pooled prevalence of GC in *H. pylori* positive patients (Prevalence: 90.90%:95% CI: 83.61–95.14), whereas Sweden had the lowest prevalence (Prevalence: 0.07%; 95% CI: 0.06–0.09). The highest and lowest prevalence was observed in prospective case series (pooled prevalence: 23.13%; 95% CI: 20.41 − 25.85; I2: 97.70%) and retrospective cohort (pooled prevalence: 1.17%; 95% CI: 0.55 − 1.78; I 2: 0.10%).

**Conclusions:**

*H. pylori* infection in GC patients varied between regions in this systematic review and meta-analysis. We observed that large amounts of GCs in developed countries are associated with *H. pylori*. Using these data, regional initiatives can be taken to prevent and eradicate *H. pylori* worldwide, thus reducing its complications.

## Background

*Helicobacter pylori* (*H. pylori*) is a bacterial pathogen associated with the gastrointestinal (GI) tract of over 50% of the world’s population [1]. *H. pylori*, is a Gram-negative spiral-shaped bacterium that colonizes the stomach, was graded as a Group I carcinogen in 1994 by the International Agency for Research on Cancer [2]. With its flagella, *H. pylori* is capable of moving and can survive on stomach acids, leading to colonization of GI tract cells and irritation and inflammation [3]. Epidemiologic and clinical data have demonstrated the role of *H. pylori* in up to 75% of non-cardia gastric malignancies and up to 98% of gastric cardia malignancies [4]. There is a strong correlation between gastric cancer (GC) and *H. pylori* infection [5].

Gastric cancer (GC) is the fifth most common cancer in the world and has the third highest mortality rates, for both sexes [6]. In 2020, actually 1.09 million new GC cases and 0.77 million deaths from GC was estimated all over the world [7]. The overall yearly incidence rates globally are 15.6 to 18.1 and 6.7 to 7.8 per 100,000 individuals in men and women, respectively [8]. According to anatomical subsites, GC can be classified into two categories: cardia GC and non-cardia GC [9]. Cardia and non-cardia GC are treated as two different diseases due to different epidemiological characteristics and distinct pathogeneses. Non-cardia GC is more common than cardia GC. Non-cardia GC accounted for up to 82% of all GC cases around the world in 2018 [10].

The high incidence of *H. pylori* infection is not always associated with high prevalence of GC. This enigma of *H. pylori* infection and GC, defined by a very high incidence of infection but a low rate of GC, was first described by Holcombe in 1992 as the "African Enigma" [11]. Hence, the African enigma represents a modification of the inflammatory response triggered by the infection, leading to the absence of any neoplastic manifestations [11]. Other countries including China, Colombia, India, Costa Rica, and Malaysia have described similar enigmas [11]. Several previous studies have suggested that an increased risk of GC is associated with lifestyle behaviors, such as cigarette smoking, intensive alcohol consumption, high salt intake, consumption of processed meat, and low intake of fruits [12]. In addition, host’s genetics has been associated with GC. Mutation in CDH1 gene that encodes E-cadherin protein for cell–cell adhesion has been associated with more than 80% increased risk of GC, and patients with reduced expression of the E-cadherin protein have a poor prognosis [13].

The majority of infections are asymptomatic, therefore a screening and treatment program cannot be justified except for high-risk patients [14]. However, the inflammatory response to an infection in a host and the virulence of the infection vary between individuals. Additionally, environmental exposures may also contribute to the increase in the risk of GCs [15]. Infection prevalence shows large geographical variations. In general, the prevalence of infection is higher in developing countries than developed countries such as Europe and North America [16]. Despite the global prevalence of GC in people with *H. pylori* infection was reported by Pormohammad et al. [1], a complete up-to-date research on the prevalence of GC in people with *H. pylori* infection has not been done yet. In the previous study, only studies conducted until 2016 were evaluated. However, in this review, statistics until 2021 were considered. Also, there were several differences between 2 studies in terms of the data bases, time period of search, eligibility criteria, and keywords. Hence, this study aimed to update the GC estimate in *H. pylori* positive patients after reviewing existing evidence and reassessing the global burden of GC caused by *H. pylori* in different regions.

## Methods

### Search strategy

PubMed, Web of Science, and Embase were searched from1 January 2011 to 20 April 2021 to retrieve all relevant studies in the world. MeSH keywords and search strategy were as below: 'Stomach Neoplasm' [tiab], OR 'Cancer of Stomach' [tiab], OR 'Gastric Cancer' [tiab], OR 'Cancer of Gastric' [tiab], OR '' Stomach Cancer '[tiab], OR 'Neoplasm of Stomach' [tiab] AND '*Helicobacter pylori*' [tiab], OR '*Campylobacter pylori'* [tiab], OR *'Campylobacter pylori* subsp. *pylori*' [tiab] OR, *'Campylobacter pyloridis'* [tiab], OR *'Helicobacter nemestrinae'* [tiab] AND 'Prevalence' [tiab], OR 'Frequency' [tiab].

### Eligibility criteria

We set our inclusion and exclusion criteria based on PECOTS criteria (population, exposure, comparison, outcome, time and study design) (Table 1). For that, all cross-sectional, prospective and retrospective case-series studies which reported the prevalence of GC in *H. pylori* patients were included. However, case reports and case series with less than five patients (as study population) and also clinical trial studies were excluded. Also, studies without reported prevalence data, definite sample sizes, and clear correct estimates of the prevalence, as well as case–control studies and abstracts presented in scientific meetings with no sufficient data were excluded from this study.

**Table 1:** PECOTS criteria of the study

**Selection criteria**	**Inclusion criteria**	**Exclusion criteria**
**Population**	Patients that have gastric cancer that diagnosed using invasive or non-invasive criteria, including endoscopy, pathology, histology, fiberscopy. PET/CT imaging, immunohistochemistry staining, biopsy and other methods	-
**Exposure**	Patients that have *H.pylori* that diagnosed using UT, PCR, ELISA, salt tolerance, Gram's stain, cagA gene pcr and other methods	-
**Comparison**	----	---
**Outcome**	Prevalence of cancer in positive H.pylori patients	---
**Time**	Published form 2011 to 20 April 2021	---
**Study design**	Observational studies including prospective or retrospective case series, cohort and cross sectional studies	Case control, ecological studies, in vivo studies, experimental of interventional studies, case report, lack of access to full text articles, review articles, letter to editor

### Study selection

There were 17,438 results from the initial search. Two authors (SK and RP) separately assessed these papers' eligibility, and any discrepancies were settled by consensus. The following step involved excluding 5380 duplicate articles. Also, after reviewing the titles and abstracts of the remaining publications, 11,058 papers were omitted. Of the remaining 1053 articles, 904 ineligible articles were omitted during the review of the entire texts. Eventually, 149 articles that qualified for inclusion were examined.

### Quality assessment

Newcastle Ottawa scale (NOS) was used to measure the quality of studies (Table 2). This scale is used to measure the quality of observational studies including cohort, cross-sectional and case series studies. The validity and reliability of this tool have been proven in various studies [17, 18].

**Table 2:** Quality assessment of studies by Newcastle Ottawa Scale (NOS) checklist

Author	Study design	Selection				Comparability	Outcome	
		Representativeness of the sample	Sample size	Non-respondents	Ascertainment of the exposure (risk factor)		Assessment of outcome	Statistical test
Dabiri et al. [19]	CS	*	-	*	**	NA	**	*
Taghvaei et al. [20]	CS	*	-	*	*	NA	**	*
Wang et al. [21]	CS	*	*	*	*	NA	**	*
Yakoob et al. [22]	CS	*	*	*	**	NA	**	*
Yang et al. [23]	CS	*	-	*	**	NA	*	*
Gucin et al. [24]	CS	*	-	*	**	NA	*	*
Shrestha et al. [25]	CS	*	-	*	**	NA	*	*
Ouyang et al. [26]	CS	*	*	*	**	NA	*	*
Kim et al. [27]	CS	*	-	*	**	NA	**	*
Shukla et al. [28]	CS	*	-	*	**	NA	**	*
Cherati et al. [29]	CS	*	-	*	**	NA	**	*
Raei et al. [30]	CS	*	-	*	**	NA	**	*
Abdi et al. [31]	CS	*	-	*	*	NA	**	*
Goudarzi et al. [32]	CS	*	-	*	*	NA	**	*
Al-Sabary et al. [33]	CS	-	-	*	**	NA	**	*
Ranjbar et al. [34]	CS	-	-	*	**	NA	**	*
Yadegar et al. [35]	CS	*	-	*	**	NA	**	*
Kupcinskas et al. [36]	CS	*	*	*	*	NA	**	*
Oh et al. [37]	CS	-	-	*	*	NA	*	*
Wang et al. [38]	CS	-	-	*	**	NA	*	*
Sakitani et al. [39]	CS	*	-	*	**	NA	*	*
Pakbaz et al. [40]	CS	*	-	*	**	NA	*	*
Sedarat et al. [41]	CS	*	-	*	**	NA	**	*
Shadman et al. [42]	CS	*	*	*	**	NA	**	*
Shin et al. [43]	CS	*	-	*	**	NA	*	*
Archampong et al. [44]	CS	-	-	*	**	NA	*	*
Xie et al. [45]	CS	-	-	*	**	NA	**	*
Deng et al. [46]	CS	*	-	*	**	NA	**	*
Shi et al. [47]	CS	*	-	*	**	NA	**	*
Yu et al. [48]	CS	*	-	*	**	NA	**	*
Szkaradkiewicz et al. [49]	CS	*	-	*	**	NA	**	*
Taghizadeh et al. [50]	CS	*	-	*	**	NA	**	*
Vilar e Silva et al. [51]	CS	*	-	*	**	NA	**	*
Gantuya et al. [52]	CS	*	*	*	**	NA	**	*
Shukla et al. [53]	CS	*	-	*	**	NA	**	*
Hu et al. [54]	CS	-	-	*	**	NA	**	*
Tahara et al. [55]	CS	-	-	*	*	NA	**	*
Ono et al. [56]	CS	*	-	*	*	NA	*	*
Pandey et al. [57]	CS	*	-	*	*	NA	**	*
Huang et al. [58]	CS	*	-	*	*	NA	**	*
Xie et al. [59]	CS	*	-	*	**	NA	**	*
Nam et al. [60]	CS	*	-	*	**	NA	**	*
Saber et al. [61]	CS	*	-	*	**	NA	**	*
Matsunari et al. [62]	CS	*	-	*	**	NA	*	*
Khatoon et al. [63]	CS	-	-	*	**	NA	**	*
Amiri et al. [64]	CS	-	-	*	**	NA	**	*
Farajzadeh Sheikh et al. [65]	CS	*	-	*	**	NA	**	*
El Khadir et al. [66]	CS	*	-	*	**	NA	**	*
Park et al. [67]	CS	*	-	*	*	NA	**	*
Yoon et al. [68]	CS	*	-	*	*	NA	*	*
Guo et al. [69]	CS	*	-	*	*	NA	**	*
Haddadi et al. [70]	CS	*	-	*	*	NA	**	*
Khan et al. [71]	CS	*	-	*	**	NA	**	*
Santos et al. [72]	CS	*	-	*	**	NA	**	*
GholizadeTobnagh et al. [73]	CS	*	-	*	**	NA	*	*
Toyoda et al. [74]	CS	*	-	*	**	NA	**	*
Thirunavukkarasu et al. [75]	CS	-	-	*	**	NA	**	*
Bakhti et al. [76]	CS	-	-	*	**	NA	**	*
Vannarath et al. [77]	CS	*	-	*	**	NA	*	*
Wei et al. [78]	CS	*	-	*	**	NA	**	*
Abu-Taleb et al. [79]	CS	*	-	*	**	NA	**	*
Chomvarin et al. [80]	CS	*	-	*	**	NA	**	*
Bilgiç et al. [81]	CS	*	-	*	**	NA	*	*
Abadi et al. [82]	CS	-	-	*	**	NA	**	*
Abadi et al. [83]	CS	*	-	*	**	NA	**	*
Ohkusa et al. [84]	CS	*	-	*	**	NA	**	*
Herrera et al. [85]	CS	*	-	*	**	NA	*	*
Tanaka et al. [86]	CS	*	-	*	**	NA	**	*
Choi et al. [87]	CS	*	-	*	**	NA	**	*
Masoumi Asl et al. [88]	HBS							
Vinagre et al. [89]	HBS							
Khatoon et al. [90]	HBS							

*NA* Not applicable, *CS* cross sectional

As mentioned in the methods section, the Newcastle–Ottawa Scale (NOS) consists of three domains. The first domain is Selection, which includes four items: Representativeness of the sample, Sample size, Non-respondents, Ascertainment of the exposure. If the first three items are established, one star is assigned. If the fourth item is also established, one or two stars are assigned. If none of the items are established, no star is assigned. The second domain is Comparability, which has one item: Comparability of the groups. If this item is established, one star is assigned. If it is not established, no star is assigned. The third domain is Outcome, which includes two items: Assessment of the outcome, Statistical test. If the first item is established, one or two stars are assigned. If the second item is also established, one star is assigned

### Data extraction

Two authors independently performed the study selection and validity assessment and resolved any disagreements by consulting a third researcher. First author, country, enrollment time, published time, type of study, number of *Hp*^+^ patients, mean age in *Hp*^+^ patients, detection method of *Hp*, number of patients with cancer, sort (name) of cancer, diagnosis method of GC, and prevalence (95% CI) were extracted from articles.

### Statistical analysis

All statistical tests in this study were performed with Stata 14.0. As previous researches [91, 92] the sample size, the number of patients with *H. pylori*, number of cancer cases in patient with *H. pylori*, and prevalence of GC in *H. pylori* positive patients were extracted. We applied Cochran's Q test to determine the heterogeneity. We also quantified it with the I^2^ index. Based on the Higgins classification approach, I^2^ values above 0.7 were determined as high heterogeneity. We used random effects model to estimate pooled values where that heterogeneity was high. Also we used the subgroup analysis and meta-regression analysis to find out the heterogeneity sources. Metaprop package were used to calculate the pooled prevalence with 95% confidence interval. Random-effects model was applied to estimate the pooled prevalence. This package applies double arcsine transformations to stabilize the variance in the meta-analyses. The effects of publication time, continents, age mean, sample size and study design on the studies heterogeneity were analyzed by univariate and multiple meta-regression analysis. Publication bias evaluated by “metabias” command. In case of any publication bias, we adjusted the prevalence rate with “metatrim” command applying trim-and-fill approach. Statistical significance was considered 0.05.

## Result

**Fig. 1:**
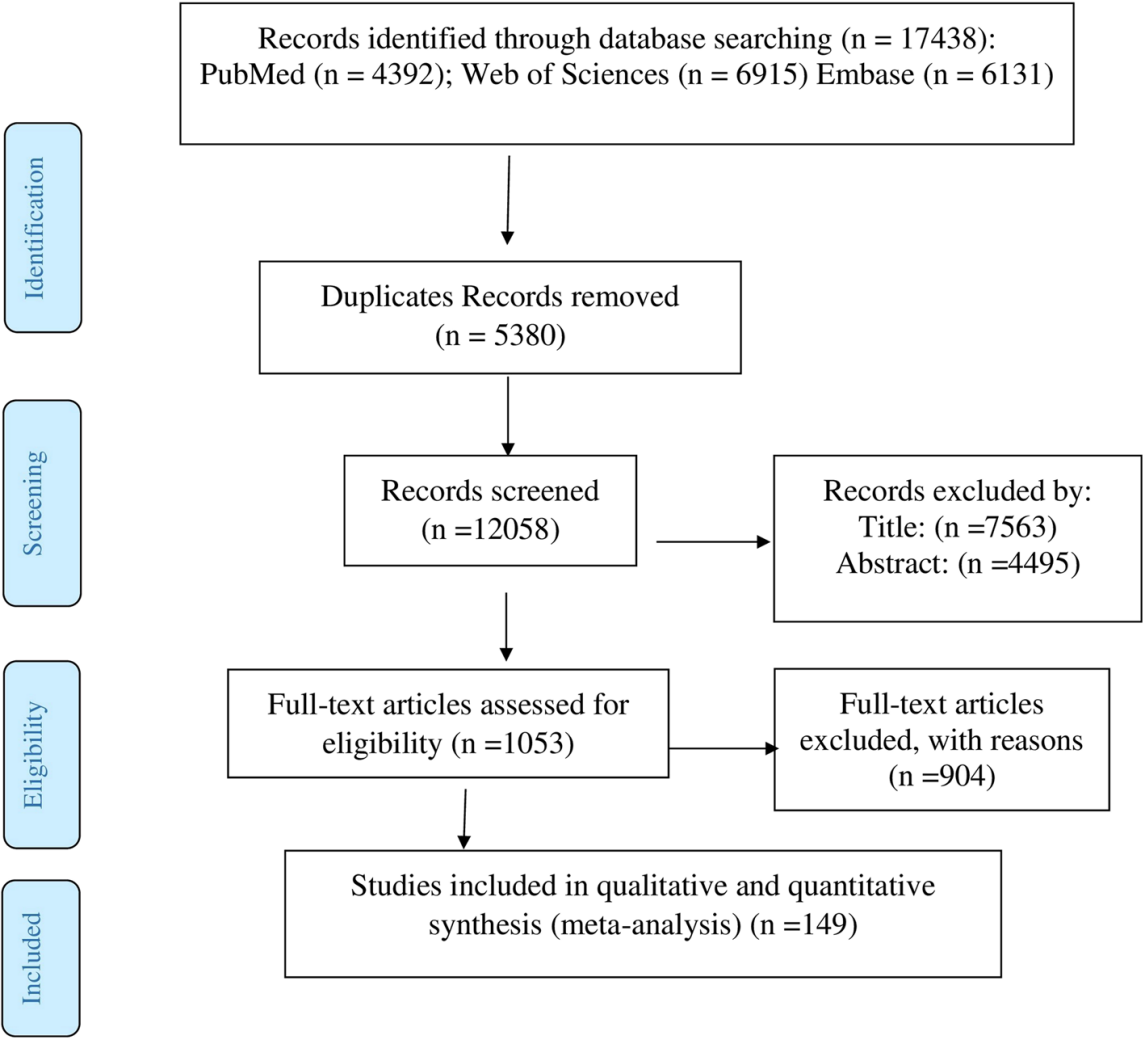
Flow diagram of study selection

A total of 149 studies with 352,872 total sample size were included in our study. Selection process flow chart is available in Fig. 1, and Table 3 shows the studies’ characteristics such as first author, country, published time and type of study. Several primary studies reported overall number of gastric cancer and do not present more detail about cancer. But some primary studies presented more detail about cancer such as anatomical location of it. Many studies mentioned they used histopathology method to detection of cancer. The highest studies number belonged to Asia continent (114 studies) area and Africa continent (6 studies) was the lowest one. All the included studies were published during 1 January 2011 to 20 April 2021. The minimum and maximum age range of the subjects was for Haddadi et al. [93] article with the age ranges (mean age = 26 years old) and Shibukawa et al. [94] study with the mean age = 73 years old, respectively. Sixty-nine (46.31%) of studies were cross sectional, sixty-four (42.95%) of studies were case series and sixteen (10.73%) of studies were cohort.

**Table 3:** Characteristics of studies included in the meta-analysis

First author	Country	Enrollment time	Published time	Type of study	Number of *Hp* + patients	Mean age in *Hp* + patients	Detection method of *Hp*	Number of patients with cancer	Sort (name) of cancer	Diagnosis method of GC	Prevalence (95% CI)
Masoumi Asl et al. [95]	Iran	March- September 2019	2020	HBS	74	53.45	UT, Histology, PCR	24	GC	Endoscopy, Histopathology	32.43 (22 to 44.32)
Khan et al. [96]	Pakistan	2005–2008	2013	CS	201	38	PCR	5	GC	Clinical diagnosis, endoscopic, histology	2.49 (0.81 to 5.71)
Santos et al. [97]	Brazil	-	2012	CS	176	59.2	RUT, histology, PCR	64	GC	Histopathological	36.36 (29.26 to 43.94)
GholizadeTobnagh et al. [98]	Iran	2007–2014	2017	CS	211	26.56	Culture, PCR, UT	38	Cardia cancer:14/38, non cardia cancer 23/38, cardia and non cardia GC:1/38; and intestinal type: 20/38 and diffuse type: 18/38	Histopathological	18.01 (13.07 to 23.87)
Toyoda et al. [88]	Japan	2004–2007	2012	CS	923	59.7	ELISA	8	Adenocarcinoma	Histopathological	0.87 (0.37 to 1.7)
Thirunavukkarasu et al. [71]	India	2011	2017	CS	62	39.68	Culture, UT, salt tolerance	19	GC	-	30.65 (19.56 to 43.65)
Cremniter et al. [72]	France	2011–2014	2018	Prospective cohort	183	56	Culture, real-time PCR	2	47:precancerous, 23:cancerous lesions, 3:atrophies, 19: metaplasias, 3:dysplasias, 2: gastric adenocarcinomas	Histopathological	1.09 (0.13 to 3.89)
Eun Bae et al. [73]	Korea	2005–2016	2018	Retrospective cohort	19,754	48	Serologic test	106	GC	Endoscopy	0.54 (0.44 to 0.65)
Bakhti et al. [74]	Iran	2019–2020	2020	CS	290	46.52	UT, Gram's stain, positive catalase, urease and oxidase tests, culture, histology, PCR	89	89: GC, 38cardia GC, 47:non-cardia GC, 4: both the types of cardia GC and non-cardia GC. 57: intestinal- type adenocarcinoma, 25: diffuse-type adenocarcinomas, 7: other pathologic types of cancer	Endoscopic and histopathologic tests	30.69 (25.43 to 36.35)
Vannarath et al. [75]	Laos	2010–2012	2014	CS	119	46	RUT, PCR	3	GC	Histological	2.52 (0.52 to 7.19)
Wei et al. [99]	China	2007–2008	2012	CS	197	49.67	Histology, PCR	53	GC	Pathological	26.9 (20.85 to 33.67)
Dabiri et al. [100]	Iran	February—June 2014	2017	CS	160	45.5	Culture, PCR	15	GC	-	9.38 (5.34 to 14.99)
Taghvaei et al. [76]	Iran	2007–2010	2011	CS	140	41.5	PCR, RUT	32	GC	Endoscopic and pathologic	22.86 (16.19 to 30.71)
Raza et al. [77]	Pakistan		2020	PCS	147	**-**	PCR	34	GC	HE, modified Giemsa stain	23.13 (16.58 to 30.79)
Wang et al. [78]	China	May- September 2010	2014	CS	80	-	RUT, Geimsa staining	10	GC	IHC	12.5 (6.16 to 21.79)
Dadashzadeh et al. [19]	Iran	2016	2017	PCS	109	39	Culture, PCR	9	GC	-	8.26 (3.85 to 15.1)
Yakoob et al. [20]	Pakistan	2013–2014	2016	CS	309	45	RUT, histology, PCR Culture	54	GC	Histopathology	17.48 (13.41 to 22.18)
Sonnenberg et al. [101]	USA	2008–2011	2013	PCS	16,759	59.2	IHC	172	Adenocarcinoma	Colonoscopy and EGD histopathological analysis:	1.03 (0.88 to 1.19)
Yang et al. [21]	China	2015–2017	2018	CS	59	58.9	UBT, IHC	9	GC	Biopsy	15.25 (7.22 to 26.99)
Vinagre et al. [102]	Brazil	2013–2014	2015	HBS	506		PCR	145	GC	Histopathological analysis: HE staining	28.66 (24.75 to 32.81)
Li et al. [22]	China	-	2020	PCS	160	53.2	RUT, IHC	75	Adenocarcinoma	Histopathological	46.88 (38.95 to 54.92)
Gucin et al. [103]	Turkey	2007–2011	2013	CS	66	-	RUT, PCR	35	GC	IHC analysis, apoptosis assays, TUNEL assay Histopathology	53.03 (40.34 to 65.44)
Pandey et al. [23]	India	-	2014	PCS	543		RUT, Histology	10	GC	-	1.84 (0.89 to 3.36)
Shrestha et al. [89]	Nepal	2011- 2013	2014	CS	155	44.7	HE, Geimsa staining	3	GC	Endoscopy	1.94 (0.4 to 5.55)
Sheikhani et al. [104]	Iraq	2007–2008	2010	PCS	54	43.22	HE staining, Modified Giemsa stain, ELISA	6	GC	Histopathology	11.11 (4.19 to 22.63)
Ouyang et al. [24]	China	2007–2012	2021	CS	79	-	RUT, Giemsa staining	22	GC	Pathology findings	27.85 (18.35 to 39.07)
Leylabadlo et al. [105]	Iran	-	2016	PCS	88	-	Culture, PCR	26	GC	Endoscopic and pathology	29.55 (20.29 to 40.22)
Alaoui Boukhris et al. [25]	Morocco	2009—2013	2013	PCS	478	-	PCR	25	Signet ring cell carcinoma (20/48), adenocarcinoma (18/48), MALT lymphoma (10/48)	Histopathology	5.23 (3.41 to 7.62)
Khamis et al. [106]	Iraq	-	2018	PCS	194	48	RUT, culture, histology examination, PCR	77	GC	Endoscopy	39.69 (32.75 to 46.95)
Gunaletchumy et al. [26]	Malaysia	-	2014	PCS	27	-	-	4	GC	Endoscopic and histological examinations	14.81 (4.19 to 33.73)
Doorakkers et al. [107]	Sweden	2005 -2012	2018	Cohort	95,176	60.1	-	75	Gastric adenocarcinoma: 75 Non-cardia gastric adenocarcinoma: 69 Cardia adenocarcinoma: 6	-	0.08 (0.06 to 0.1)
Horie et al. [108]	Japan	2005–2018	2020	Retrospective	1300	58.3	-	37	GC	-	2.85 (2.01 to 3.9)
Kim et al. [109]	Korea	February 2006 and July 2015	2020	CS	137	54.9	Giemsa staining, CLO test, culturing, serology	69	GC	-	50.36 (41.7 to 59.01)
Shukla et al. [110]	India	2007& 2010	2012	CS	105	46.34	RUT, culture, histopathology, PCR	24	GC	Clinical, endoscopic, and histopathological examination	22.86 (15.23 to 32.07)
Cherati et al. [111]	Iran	Mar 2015 and September 2015	2017	CS	67	52.2	PCR	28	GC	Histologically	41.79 (29.85 to 54.48)
El Khadir et al. [112]	Morocco	-	2018		827		PCR	81	GC	Histopathological examination	9.79 (7.85 to 12.03)
Raei et al. [27]	Iran	2007 to 2014	2015	CS	242		Culture, PCR	42	Cardia cancer:18/42 Non-cardia cancer:24/42 Intestinal-type adenocarcinoma:24/42 Diffuse-type adenocarcinoma:16/42 Invasive squamous cell-type carcinoma:1/42 Mucin producing-type adenocarcinoma:1/42	Histopathological examination	17.36 (12.8 to 22.73)
Abdi et al. [28]	Iran	2012–2014	2016	CS	83	48.7	PCR	27	GC	Histopathological	32.53 (22.65 to 43.7)
Ansari et al. [29]	Bhutan, Myanmar, Nepal and Bangladesh	2010–2014	2017	PCS	374	37.9	PCR, histological	5	GC	Endoscopic examination/ histopathological method	1.34 (0.44 to 3.09)
Ortiz et al. [113]	USA	2013	2019	PCS	116	52	Culture, PCR	23	Adenocarcinoma Diffuse:10Intestinal:12 Mixed:1	Histopathologic diagnoses	19.83 (13 to 28.25)
Mohammadi et al. [30]	Iran	-	2019	PCS	120	52	PCR	11	GC	Endoscopy	9.17 (4.67 to 15.81)
Yeh et al. [31]	Taiwan	-	2019	PCS	164	59.2	H&E, modified Giemsa stains, PCR, ELISA	30	GC	Histological	18.29 (12.7 to 25.07)
Sheu et al. [114]	Taiwan	-	2012	PCS	92		Histology, cultures	20	GC	Endoscopy with histological confirmation	21.74 (13.81 to 31.56)
Yeh et al. [115]	Taiwan	2009–2010	2011	Prospective	145	49.3	Histology and cultures	22	GC	Endoscopy	15.17 (9.76 to 22.07)
Goudarzi et al. [116]	Iran	2012- 2013	2015	CS	98	49	Culture, RUT	35	GC	-	35.71 (26.29 to 46.03)
Phan et al. [117]	Vietnam	2012–2014	2017	PCS	96	44.1	Culture, PCR	2	GC	Histology	2.08 (0.25 to 7.32)
Al-Sabary et al. [118]	Iraq	Feb to Sep 2016	2017	CS	92	-	Culture, PCR	3	GC	Endoscopy	3.26 (0.68 to 9.23)
Ranjbar et al. [119]	Iran	2016-2017	2018	CS	526	-	Cultured, histology	4	Gastric:4	Endoscopy	0.76 (0.21 to 1.94)
Hernandez et al. [32]	Mexico	-	2018	PCS	307	-	ELISA	87	GC	Histology	28.34 (23.37 to 33.74)
Blanchard et al. [120]	Multi-country *	-	2013	PCS	65	-	-	4	GC	-	6.15 (1.7 to 15.01)
Zeng et al. [33]	China	1994 and 2002	2011	Cohort	967	-	ELISA, Serology	160	GC 109: intestinal, 104: diffuse, and 35: mixed type	Histopathologic diagnosis	16.55 (14.26 to 19.04)
Boonyanugomol et al. [34]	Thailand and Korea	-	2020	PCS	170	-	RUT-Culture -PCR	40	GC	Endoscopy	23.53 (17.37 to 30.63)
Ogawa et al. [121]	Japan	-	2017	PCS	43	-	Culture	10	GC	Endoscopy	23.26 (11.76 to 38.63)
Boonyanugomol et al. [122]	Thailand	-	2019	PCS	80	-	RUT, PCR	10	GC	Endoscopy	12.5 (6.16 to 21.79)
Ghoshal et al. [123]	India	-	2014	PCS	68	54.3	RUT, histology, ELISA	21	GC	Histology, Endoscopy, Surgery	30.88 (20.24 to 43.26)
Farzi et al. [124]	Iran	-	2018	PCS	68	47	Culture, PCR	5	GC	Endoscopic and pathological findings	7.35 (2.43 to 16.33)
Yadegar et al. [125]	Iran	2011–2012	2019	CS	61	36	Culture, PCR	5	GC	Histopathological examination	8.2 (2.72 to 18.1)
Hashemi et al. [126]	Iran	2015–2016	2019		157	-	PCR, Culture, UT	22	GC	endoscopy	14.01 (8.99 to 20.44)
Kupcinskas et al. [127]	Germany	2005–2012	2014	CS	477	-	Serology	191	GC Intestinal:136, Diffuse:89 Mixed:33, Data unavailable:105	Histological subtyping of GC: Laurén classification into intestinal and diffuse-types	40.04 (35.61 to 44.59)
Shibukawa et al. [94]	Japan	2006–2019	2021	Retrospective	1003	74	Serological testing, RUT, IHC, SAT	168	GC	Endoscopic characteristics	16.75 (14.49 to 19.21)
Oh et al. [128]	Korea	2008–2013	2019	CS	187	-	Warthin-Starry silver impregnation method	35	GC	-	18.72 (13.4 to 25.06)
Wang et al. [35]	China	2015–2018	2020	CS	61	55.9	Giemsa staining method	32	Non-cardia gastric adenocarcinoma	Histologically	52.46 (39.27 to 65.4)
Boreiri et al. [129]	Iran	2000–2001	2013	Cohort	892	53.1	RUT	32	GC	Histological	3.59 (2.47 to 5.03)
Sakitani et al. [36]	Japan	January 1996 and March 2013	2015	CS	965	62.9	RUT, serological testing, UBT, pathological analysis	21	GC Intestinal type:16 Diffuse type:5	Pathology	2.18 (1.35 to 3.31)
Sekikawa et al. [37]	Japan	January 2004 and December 2012	2016	Cohort	236	-	-	14	GC	Histology, Endoscopy	Sekikawa et al. (201–-5.93 (3.28 to 9.75)
Pakbaz et al. [38]	Iran	March to August 2011	2013	CS	82	46	RUT, PCR	13	GC	Endoscopy	15.85 (8.72 to 25.58)
Sedarat et al. [130]	Iran	2013- 2015	2018	CS	150	43	RUT, PCR	4	GC	Histology, Endoscopy	2.67 (0.73 to 6.69)
Shadman et al. [39]	Iran	2011 -2012	2015	CS	133	63.2	Histopathological examination, RUT	47	GC Well, differentiated:3 Moderately differentiated:10 Poorly differentiated:15 Undifferentiated:4	Histopathological	35.34 (27.25 to 44.09)
Shin et al. [131]	korea	2006–2014	2016	CS	132	60.3	Histology, CLO test, culture	26	GC	Endoscopy and histopathology	19.7 (13.29 to 27.51)
Archampong et al. [40]	Ghana	2010& 2012	2016	CS	198	-	RUT-CLO	19	GC	Endoscopy and histopathology	9.6 (5.88 to 14.58)
Kobayashi et al. [41]	Japan	April 2005 & November 2015	2016	Retrospective	37	-	RUT, SAT	7	Early gastric cancer:4 Gastric adenoma:2 MALT lymphoma:1 Other fiberscopic findings: 3	Fiberscopy. PET/CT imaging	18.92 (7.96 to 35.16)
Xie et al. [42]	China	2007–2008	2014	CS	142	58.3	RUT, modified Giemsa staining	61	GC Male:39, Female: 22	Pathological diagnosis	42.96 (34.69 to 51.53)
Deng et al. [43]	China	2008& 2013	2014	CS	76	-		7	Among the 176 GC cases, 63: intestinal type, 96:diffuse type, 17: mixed type	Pathological diagnosis	9.21 (3.78 to 18.06)
Shi et al. [44]	China	2010—2012	2014	CS	40	-	RUT,Warthin-Starry staining. Gram staining. Oxidase and catalase tests	13	GC: 2 tissues at an early stage and 11 tissues at an advanced stage; 6 intestinal type tissues, 4 diffuse type tissues, and 3 mixed type tissues	Pathological diagnosis	32.5 (18.57 to 49.13)
Yu et al. [132]	China	1992 -2007	2014	CS	217	59.15	IHC -PCR	116	intestinal type:97, diffuse type: 95	Histopathology	53.46 (46.58 to 60.24)
Zabaglia et al. [45]	Brazil	-	2017	PCS	72	65,6	PCR	19	GC	Histopathology	26.39 (16.7 to 38.1)
Szkaradkiewicz et al. [46]	Poland	2013–2014	2016	CS	42	65	PCR	15	GC	Histopathology	35.71 (21.55 to 51.97)
Jorge et al. [47]	Brazil	-	2013	PCS	27	63.4	Multiplex PCR	11	Intestinal: 12 Diffuse type: 8	Histopathology	40.74 (22.39 to 61.2)
Taghizadeh et al. [48]	Iran	2012 -2013	2014	CS	84	-	Histopathology, RUT	21	GC	Endoscopic, Histopathology	25 (16.19 to 35.64)
Khatoon et al. [133]	India	2012–2016	2018	HBS	122	47.34	RIT, culture, histopathology, PCR	40	Intestinal:38 Diffuse: 32	clinical, endoscopic and histopathological findings	32.79 (24.56 to 41.87)
Yan et al. [49]	China	2019–2020	2021	PCS	294	62.4	UBT, RUT, histopathology	132	GC	Endoscopy	44.9 (39.12 to 50.78)
Vilar e Silva et al. [134]	Brazil	2010- 2011	2014	CS	384	59.9	PCR	190	61/190: diffuse type 129/190: intestinal type	Histological	49.48 (44.37 to 54.6)
Anwar et al. [50]	Egypt	2008–2009	2012	PCS	40	46.9	Serological, ELISA	20	GC Intestinal:10 Diffuse: 7 Mixed: 3	History and clinical examination, Endoscopy and histopathology	50 (33.8 to 66.2)
Gantuya et al. [90]	Mongolia	2014–2016	2019	CS	606	53.8	RUT, culture, Histology, IHC, serology, updated Sydney system	27	GC	Endoscopy and histopathology	4.46 (2.96 to 6.42)
Beheshtirouy et al. [135]	Iran	2016–2018	2020	RCS	62	-	PCR	35	GC	-	56.45 (43.26 to 69.01)
Park et al. [51]	Korea	2015	2019	PCS	58	54.1	RUT, Serology, EIA, latex agglutination turbidimetric immunoassay,	32	GC	Histopathology	55.17 (41.54 to 68.26)
Shukla et al. [136]	India	2005–2009	2011	CS	118	-	RUT, Culture, histopathology, PCR	31	GC	Histopathology	26.27 (18.6 to 35.17)
Toyoshima et al. [52]	Japan	2002–2014	2017	RCS	1232	54.1	UBT, Serology, SAT	15	GC	Histological evaluation: Vienna classification	1.22 (0.68 to 2)
Spulber et al. [137]	Romania	2012–2013	2015	Retrospective cohort	1694	55	Fast urease test	46	GC	Endoscopy	2.72 (1.99 to 3.61)
Sugimoto et al. [138]	Japan	2013–2015	2017	RCS	1200	71.3	Anti-*Hp*- IgG serological test a PCR, culture UBT	268	De novo cancers: 248 metachronous cancers: 20	Endoscopy	22.33 (20.01 to 24.8)
Kobayashi et al. [53]	Japan	2013 -2017	2019	RCS	1271	61	Serum anti-H. pylori antibodies, UBT, SAT, histopathology	84	MALT:16	Histopathology	6.61 (5.31 to 8.12)
Leung et al. [139]	China	2003–2012	2018	Cohort	73,237	55.2	Endoscopy	200	GC	-	0.27 (0.24 to 0.31)
Watari et al. [140]	Japan	-	2019	Cohort	61	70	UBT, Giemsa staining, IgG antibody test	37	GC	Histological analysis	60.66 (47.31 to 72.93)
Nam et al. [141]	Korea	2003–2011	2019	Retrospective cohort	5558	52.6	RUT	46	Early GC: 29 AGCs gastric cardia: 2	Endoscopic resection	0.83 (0.61 to 1.1)
Sallas et al. [142]	Brazil	-	2019	PCS	72	65.6	PCR	19	GC	Histological classification: Sydney system	26.39 (16.7 to 38.1)
Queiroz et al. [143]	Brazil	-	2011	PCS	252	61.9	Histopathological study, PCR	58	Non-cardia gastric adenocarcinoma	Histopathology	23.02 (17.97 to 28.71)
Sun et al. [144]	China	-	2018	PCS	49	-	UBT	25	GC	Pathology: gastric resection	51.02 (36.34 to 65.58)
Jiang et al. [145]	China	2003–2012	2016	RCS	43,080	-	RUT	1497	GC	Endoscopy and histopathology	3.47 (3.3 to 3.65)
Hu et al. [146]	China	2015–2016	2019	CS	57	-	RUT, IHC	16	GC	-	28.07 (16.97 to 41.54)
Ferraz et al. [147]	Brazil	-	2015	PCS	94	40.3	PCR	44	Neoplastic:21, adjacent nonneoplastic tissue:23	Histopathology	46.81 (36.44 to 57.39)
Tahara et al. [148]	Japan	2013–2016	2019	CS	87	-	Histological analysis and molecular study	43	Metachronous:8 GC:35	Histological analysis and molecular study	49.43 (38.53 to 60.36)
Vaziri et al. [149]	Iran	-	2013	PCS	71	66	Culture, PCR	1	GC	Endoscopy and histopathology	1.41 (0.04 to 7.6)
Boonyanugomol et al. [54]	Thailand and Korea	-	2018	PCS	95	-	RUT, PCR	10	GC	-	10.53 (5.16 to 18.51)
Ono et al. [150]	Dominican	2011–2016	2020	CS	175	-	Culture, PCR	1	GC	Histopathology	0.57 (0.01 to 3.14)
Pandey et al. [55]	India	2007–2012	2018	CS	99	-	PCR, Culture	34	Diffuse‐type:44, Intestinal‐type:21	IHC	34.34 (25.09 to 44.56)
Link et al. [151]	Germany	2011–2013	2015	PCS	41	68.6	Culture rapid urease test, serology, histology and microbiology	8	Cardia:7,Corpus:6 Antrum:3,Diffuse:5 Intestinal:9,other 2	Histopathology	19.51 (8.82 to 34.87)
Casarotto et al. [152]	Italy	-	2019	PCS	91	-	Histological Study Gram staining, and urease production	39	GC	Histopathology	42.86 (32.53 to 53.66)
Zao et al. [56]	China	-	2020	PCS	177	-	Culture, PCR	33	GC	-	18.64 (13.19 to 25.17)
Abu-Taleb et al. [57]	Egypt	2016–2017	2018	CS	90	-	RUT, PCR	4	GC	Endoscopy	4.44 (1.22 to 10.99)
Chomvarin et al. [153]	Thailand		2012	CS	147	50	Gram’s staining, catalase, oxidase and UT, PCR	18	GC	-	12.24 (7.42 to 18.66)
Bilgiç et al. [154]	Turkish	2014–2015	2018	CS	95	55.71	RT-PCR	34	GC	Histopathology epigenetic assessments	35.79 (26.21 to 46.28)
Chiu et al. [155]	Taiwan		2018	Cohort	60	-	Gastric endoscopy	18	Adenocarcinoma	Endoscopy	30 (18.85 to 43.21)
Kumar et al. [79]	USA	1994–2018	2020	Cohort	36,695	60.4	Pathology, SAT, UBT	108	Oesophageal and proximal GCs	Endoscopy	0.29 (0.24 to 0.36)
Nishikawa et al. [80]	Japan	2006–2014	2018	Cohort	674		UBT, RUT, EIA	25	Gastric cancer	Endoscopy	3.71 (2.41 to 5.43)
Sadjadi et al. [81]	Iran	-	2014	Cohort	928	53.1	Histology, RUT	36	GC	Histological	3.88 (2.73 to 5.33)
Abadi et al. [156]	Iran	2009–2010	2011	CS	128	-	Culture, PCR	28	Adenocarcinoma	Endoscopy	21.88 (15.05 to 30.04)
Hnatyszyn et al. [157]	Poland	-	2013	PCS	131	36	RUT, IgG antibodies, histopathological examination	17	GC	Endoscopy and histopathology	12.98 (7.74 to 19.96)
Abadi et al. [158]	Iran	2007–2010	2012	CS	232	44	Gram staining, Acid resistance testing, Endoscopy, PCR	32	GC	Histopathology	13.79 (9.63 to 18.91)
Ohkusa et al. [159]	Japan	1994–2000	2004	CS	172	53	Endoscopy, RUT, UBT, histological examination	5	GC gastric adenoma or early cancer	Endoscopy	2.91 (0.95 to 6.65)
Abe et al. [82]	Japan	-	2010	PCS	254	56.8	Culture, IHC	28	GC	Endoscopy	11.02 (7.45 to 15.54)
Lahner et al. [160]	Italy	-	2011	PCS	29	52.5	Biopsy, immunoproteome technology	10	GC	-	34.48 (17.94 to 54.33)
Herrera et al. [83]	Mexico	1999–2002	2013	CS	137	55.3	ELISA	41	Gastric adenocarcinoma	Endoscopic and Histopathology	29.93 (22.41 to 38.34)
Tanaka et al. [161]	Japan	2003–2007	2011	CS	99	59.1	Biopsy immunoproteome technology	90	Gastric carcinoma	IHC	90.91 (83.44 to 95.76)
Batista et al. [84]	Brazil	-	2011	Cohort	436	52.7		188	GC	Endoscopy pepsinogen tests	43.12 (38.42 to 47.92)
Choi et al. [162]	South Korea	2006–2013	2015	CS	237	-	Modified Giemsa staining, culture, RUT, PCR	71	GC	Biopsy, serum pepsinogen tests	29.96 (24.2 to 36.23)
Chuang et al. [163]	Taiwan	-	2011	PCS	469	48.1	Modified Giemsa stain, SDS-PAGE	26	GC	Gastric biopsy	5.54 (3.65 to 8.02)
Cavalcante et al. [85]	Brazil	2008	2012	PCS	134	46	PCR	30	Gastric carcinoma	Histopathology	22.39 (15.64 to 30.39)
Borges et al. [164]	Brazil	-	2019	PCS	75	40.9	PCR	2	Gastric adenocarcinoma	Histopathology	2.67 (0.32 to 9.3)
Salih et al. [87]	Turkey	-	2013	PCS	66	-	Giemsa, PCR, RUT	35	34 intestinal type, 1 diffuse type	Histopathology	53.03 (40.34 to 65.44)
Huang et al. [165]	China	2012–2014	2018	CS	122	-	UBT, RUT, histopathology	65	GC	Gastroscopy/histopathology	53.28 (44.03 to 62.36)
Xie et al. [166]	China	2010–2016	2018	CS	116	-	ELISA	72	19 early, 53 advanced GC	Gastroscopy/pathological	62.07 (52.59 to 70.91)
Pereira et al. [167]	Brazil	-	2020	PCS	103	-	PCR	38	GC	Histopathological	36.89 (27.59 to 46.97)
Nam et al. [168]	Korea	2003–2013	2018	CS	17,751	-	RUT	82	GC	Gastroscopy	0.46 (0.37 to 0.57)
Saber et al. [58]	Saudi Arabia	2012–2014	2015	CS	131	-	PCR, IgG antibody/culture	43	GC	Histopathology	32.82 (24.88 to 41.57)
Matsunari et al. [59]	Japan	1993–2005	2012	CS	291	-	Culture, PCR	23	GC	Endoscopy/histological	7.9 (5.08 to 11.62)
Khatoon et al. [169]	India	2012–2016	2017	CS	122	-	RUT/culture/histology PCR	40	GC	Endoscopy	32.79 (24.56 to 41.87)
Ghoshal et al. [60]	India	-	2013	PCS	185	-	RUT/ELISA	49	GC	Endoscopy & biopsy	26.49 (20.28 to 33.46)
Amiri et al. [61]	Iran	2012–2013	2016	CS	86	-	RUT/histopathological qRT-PCR	20	GC	Histopathological	23.26 (14.82 to 33.61)
Farajzadeh Sheikh et al. [62]	Iran	2014–2015	2018	CS	201	-	PCR, Gram staining Urease test, culture	22	GC	Histopathological	10.95 (6.99 to 16.1)
El Khadir et al. [63]	Morocco	2009–2019	2021	CS	823	48.2	PCR	75	GC	Endoscopically / histological	9.11 (7.24 to 11.29)
Pandey et al. [170]	India	2007–2012	2014	PCS	99	-	PCR	34	44 diffuse/ 21 intestinal adenocarcinoma	Histological	34.34 (25.09 to 44.56)
Park et al. [64]	Korea	2008–2013	2016	CS	10,947	-	Immunoglobulin, RUT, pathology	45	GC	Histological	0.41 (0.3 to 0.55)
Kawamura et al. [65]	Japan	2007–2010	2013	PCS	139	-	RUT	61	Differentiated: 46 Undifferentiated GC: 21	Magnifying endoscopy histological	43.88 (35.49 to 52.55)
Raza et al. [66]	Pakistan	-	2017	Prospective	168	-	PCR	55	GC	Histopathological	32.74 (25.71 to 40.39)
Yoon et al. [171]	Korea	2006–2014	2019	CS	303	-	Giemsa, RUT, culture ELISA	170	GC Intestinal: 119, Diffuse: 51	Endoscopically	56.11 (50.32 to 61.77)
Santos et al. [67]	Brazil	-	2020	PCS	92	-	PCR	32	GC	Histopathological	34.78 (25.15 to 45.43)
Guo et al. [69]	China	2010–2012	2014	CS	50	-	RUT, UBT, Serology	17	GC, Intestinal:18, Diffuse: 18	Histopathological	34 (21.21 to 48.77)
Haddadi et al. [93]	Iran	2013	2015	CS	128	26	Culture, PCR	14	GC	Histopathological	10.94 (6.11 to 17.67)
Wei et al. [172]	Taiwan	-	2021	Cohort	48	69	-	43	GC	-	89.58 (77.34 to 96.53)

### Pooled prevalence of GC in *H. pylori* positive patients

**Fig. 2:**
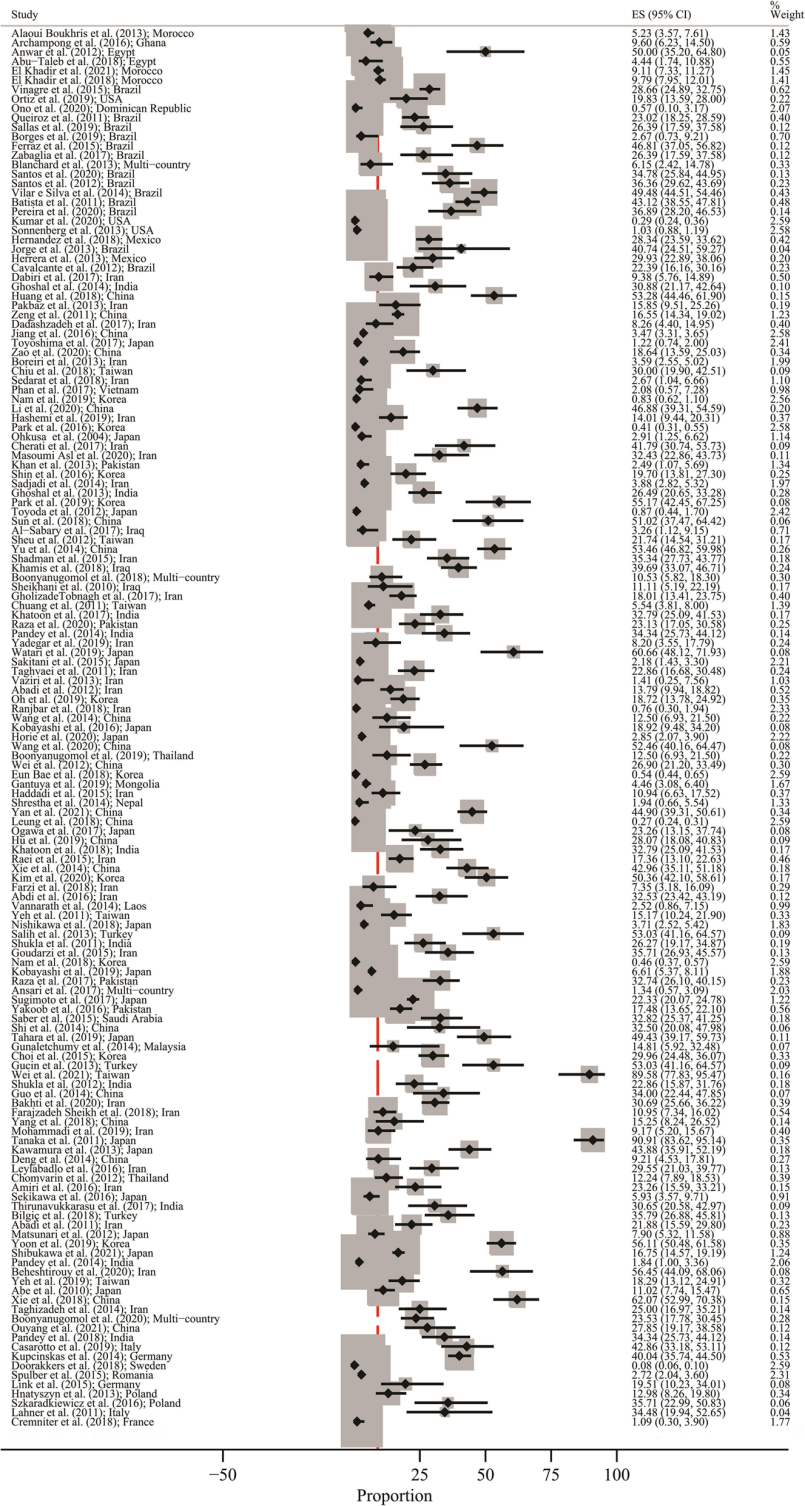
Forest plot of prevalence of gastric cancer in *Helicobacter pylori* positive patients

Figure 2 shows the forest plot of prevalence of GC in *H. pylori* positive patients. Minimum and maximum prevalence were in Doorakkers et al. [107] study (Prevalence: 0.07%; 95% CI: 0.06–0.09) from the Sweden and Tanaka et al. [161] (Prevalence: 90.90%:95% CI: 83.61–95.14) from Japan, respectively. Due to high heterogeneity and different study design, results don’t merge and presented based on different subgroups

### Pooled prevalence of gastric cancer in *H. pylori* positive patients based on different subgroups

**Fig. 3:**
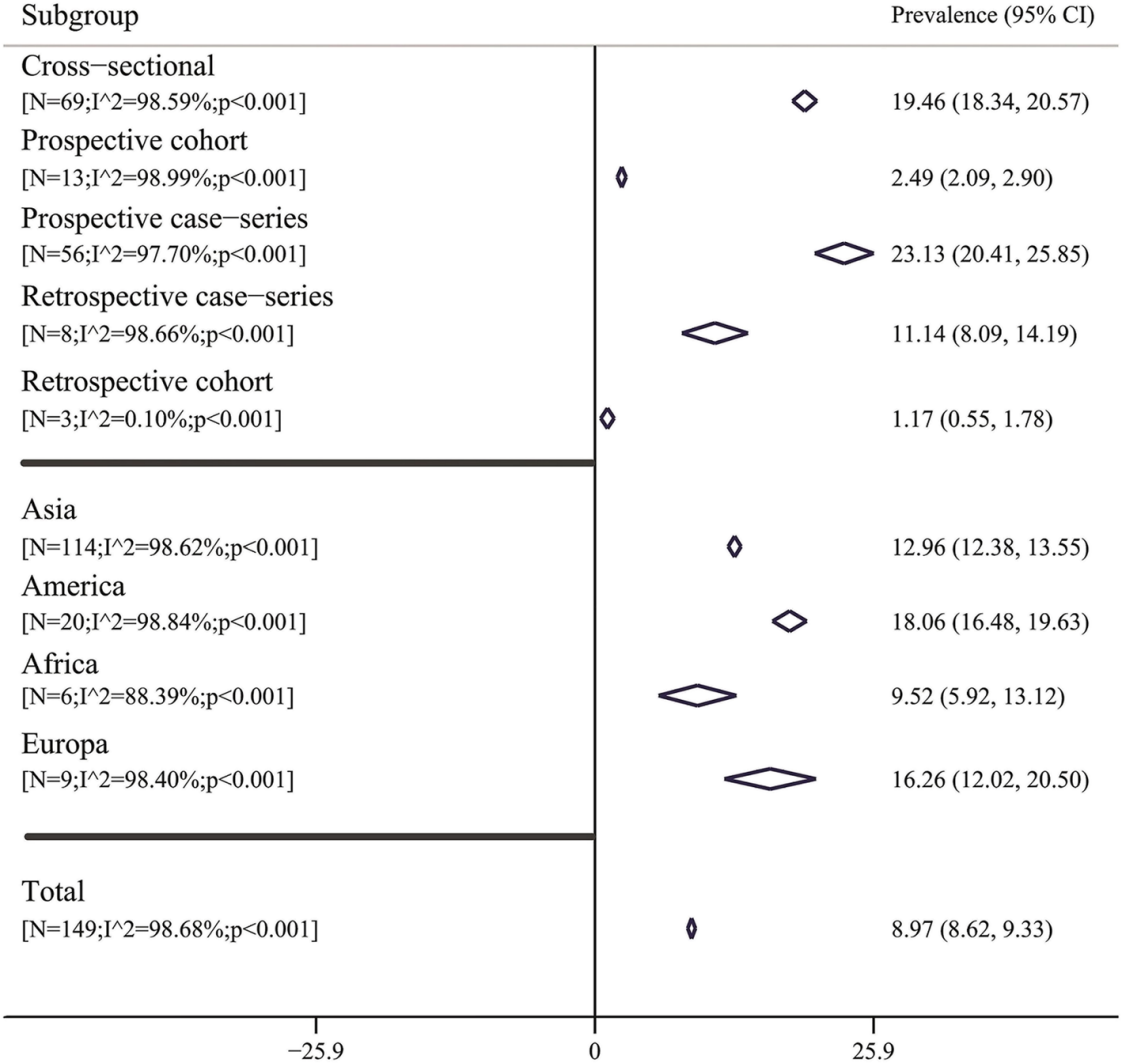
Pooled prevalence with 95% confidence interval [CI] and heterogeneity indexes of gastric cancer in *Helicobacter pylori* positive patients based on type of the design and continents places. The diamond mark illustrates the pooled prevalence and the length of the diamond indicates the 95% CI

Pooled prevalence of GC in *H. pylori* positive patients based on study design and continents are listed in Fig. 3 and Table 4. Based on design, the highest and lowest prevalence was observed in prospective case series (pooled prevalence: 23.13%; 95% CI: 20.41 − 25.85; I2: 97.70%) and retrospective cohort (pooled prevalence: 1.17%; 95% CI: 0.55 − 1.78; I 2: 0.10%), respectively. Also based on continents, the highest and lowest prevalence was observed in America (pooled prevalence: 18.06%; 95% CI: 16.48 − 19.63; I^2^: 98.84%) and Africa (pooled prevalence: 9.52%; 95% CI: 5.92 − 13.12; I^2^: 88.39%) continents, respectively.

**Table 4:** Result of meta-analysis, publication bias and fill-trim method for prevalence estimate and corresponding 95% confidence interval of gastric cancer in *H.pylori* positive patients

**Subgroup**	**Meta-analysis**			**Publication bias (Egger’s test)**		**Fill-trim method**
	**NS**	**Heterogeneity index**	**Pooled prevalence%** **(95% CI)**	**Coefficient** **(95% CI)**	***p*-value**	**Adj-pooled prevalence%** **(95% CI)**
Study design						
Cross sectional	69	I^2^ = 98.59%; *p* < 0.001	19.46 (18.34 to 20.57)	7.09 (5.82 to 8.36)	< 0.001	7.89 (6.78 to 9.01)
Prospective cohort	13	I^2^ = 98.99%; *p* < 0.001	2.49 (2.09 to 2.90)	8.59 (4.33 to 12.84)	0.001	1.13 (0.65 to 1.61)
Prospective case series	56	I^2^ = 97.70%; *p* < 0.001	23.13 (20.41 to 25.85)	6.07 (5.15 to 6.98)	< 0.001	16.23 (13.76 to 18.69)
Retrospective case series	8	I^2^ = 98.66%; *p* < 0.001	11.14 (8.09 to 14.19)	6.30 (-1.45 to 14.05)	0.094	–-
Retrospective cohort	3	I^2^ = 0.10%; *p* < 0.001	1.17 (0.55 to 1.78)	5.79 (-7.04 to 18.62)	0.110	–-
Continents						
Asia	114	I^2^ = 98.62%;* p* < 0.001	12.96 (12.38 to 13.55)	6.33 (2.03 to 10.63)	0.010	4.37 (0.03 to 8.75)
America	20	I^2^ = 98.84%; *p* < 0.001	18.06 (16.48 to19.63)	6.89 (5.87 to 7.92)	< 0.001	6.43 (7.02 to 21.43)
Africa	6	I^2^ = 88.39%; *p* < 0.001	9.52 (5.92 to 13.12)	3.41 (-3.42 to 10.26)	0.239	–-
Europa	9	I^2^ = 98.40%; *p* < 0.001	16.26 (12.02 to 20.50)	8.09 (5.54 to 10.64)	< 0.001	7.10 (5.58 to 8.63)

*CI* Confidence interval, *NS* Number of studies

### Heterogeneity and meta‐regression

**Fig. 4:**
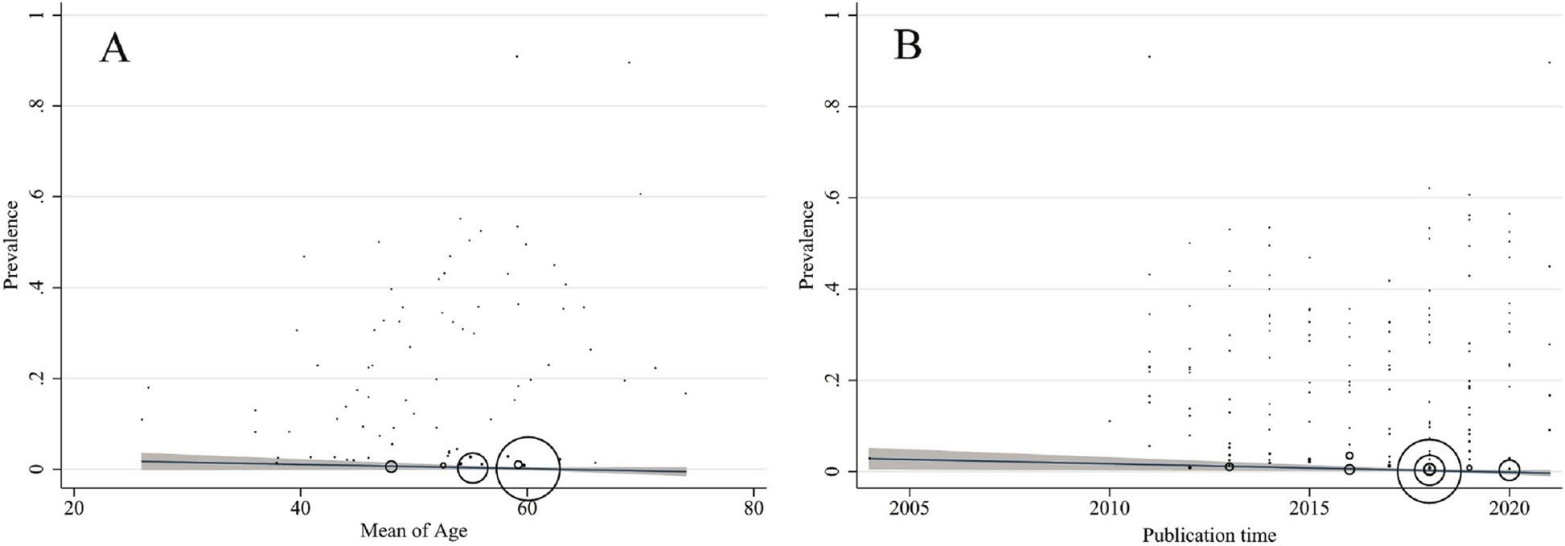
Association between Pooled prevalence of gastric cancer in *Helicobacter pylori* positive patients with age (**A**) and publication year (**B**) by means of meta-regression. The size of circles indicates the precision of each study. There is a positive significant association with respect to the pooled prevalence with age

Heterogeneity results are available in Table 4. Cochran's Q test showed the included studies had high heterogeneity (*p* < 0.001). The I^2^ index for total prevalence was up to 98%. The result of univariate meta‐regression analysis (Table 5) showed the age (Coefficient: 0.59; p: 0.009), sample size (Coefficient: − 0.1; p: 0.003) and study design (based WHO regional office) (Coefficient: 3.72; p: 0.015) possess significant effect on the studies heterogeneity (Fig. 4A and B) and have eligible to include to multiple model. The result of multiple meta‐regression analysis showed the just age (Coefficient: 0.66; p: 0.003) have a significant effect on the studies heterogeneity. The R^2^-adj for multiple model was 13.63% and this mean the age, Sample size and study design explained the about 14% of total heterogeneity of prevalence.

**Table 5:** The univariate and multiple meta-regression analysis on the determinant heterogeneity in effect of iron therapy on depression

**Variables**	**Univariate meta-regression**			**Multiple meta-regression**		
	**Coefficient (95% CI)**	***p*-value**	***R***^**2**^**-adj**	**Coefficient (95% CI)**	***p*-value**	***R***^**2**^**-adj**
Publication time (yrs.)	0.43 (-0.53 to 1.39)	0.382	0.1%	Not included	–-	13.63%
Continents (score)	1.60 (-2.43 to 5.63)	0.433	0.02%	Not included	–-	
Age mean (yrs.)	0.59 (0.15 to 1.03)	0.009	6.96%	0.66 (0.24 to 1.08)	0.003	
Sample size (number)	-0.01 (-0.02 to -0.01)	0.003	5.41%	0.-01 (-0.2 to 0.01)	0.84	
Study design (score)	3.72 (0.73 to 6.71)	0.015	3.49%	3.08 (-0.92 to 7.08)	0.130	

*CI* Confidence Interval, Coding of study design: 1 = Retrospective cohort; 2 = Prospective cohort; 3 = Retrospective case-series; 4 = Cross sectional; 5 = Prospective case-series

Coding of study continent: 1 = Africa; 2 = America; 3 = Asia; 4 = Europa

^*^Significant at 0.05

### Publication bias

The results of Egger’s test showed significant publication bias in our meta-analysis which provided in Table 4. For adjustment of pooled prevalence, fill and trim method was used that result was showed in Table 4. Based on this result, publication-bias-adjusted pooled prevalence estimation for cross sectional was 7.89% (95% CI: 6.78—9.01) which was different with pooled prevalence estimation based on meta-analysis 19.46% (95% CI: 18.34 to 20.57). Result of fill and trim method for other subgroups was showed in Table 4.

## Discussion

Infection with *H. pylori* causes chronic inflammation and significantly increases the risk of developing duodenal and gastric ulcer disease and GC. *H. pylori* primarily infect the epithelial cells in the stomach and can survive in humans for decades by inhibiting the immune system responsiveness, results inducing chronic inflammatory responses. Because of endotoxin elaboration and other inflammatory exudates, the colonization of the gastric mucosa by *H. pylori* has been observed with gastric atrophy [173]. Researchers have recently reported molecular aspects that highlight the importance of certain apoptotic genes and proteins including C-Myc, P53, Bcl2, and Rb-suppressor systems in *H. pylori* pathogenesis. *H. pylori* infection has also been shown to be related to nitric oxide (NOSi genotype) [70]. Induction of apoptosis in gastric mucosa by *H. pylori* involves upregulation of Bax and Bcl-2 [70].

With *H. pylori* involvement in the gastric intestinal pH alteration, dysplasia has been observed in patients with *H. pylori* infection [174]. Previous studies have been shown that individuals who had been infected with *H. pylori* were six times more likely to develop GC compared with healthy people [175]. In this study, using random-effects model approach, pooled prevalence of GC in *H. pylori* positive patients was 8.97% (95% CI: 8.62–9.33) [*N* = 149; I^2^ = 98.68%]. Therefore, from every 1000 *H. pylori* positive patients, 8.62 to 9.33 individuals get GC. The frequency of *H. pylori* in people less than 50 years old was reported as 41.9%.

The study by Vohlonen et al. showed risk ratio (RR) of stomach cancer in people with *H. pylori* infection was 5.8 (95%CI: 2.7–15.3) compared to people with healthy stomachs, and 9.1 (95%, CI: 2.9–30.0) in men with atrophic gastritis [86]. The present observation also demonstrated that an *H. pylori* infection alone (non-atrophic *H. pylori* gastritis) is by itself a clear risk condition for GC as was suggested by the IARC/WHO statement in 1994 [176]. In study conducted before 1998, by approximately 800 GC cases, the analysis yielded a risk ratios of 2.5 (95% CI: 1.9–3.4) for GC in *H. pylori*-seropositive people [177]. Another study including 233 GCs and 910 controls, yielded a risk ratios of 6.5 (95%CI: 3.3–12.6) for non-cardia GC in subjects infected with a cytotoxic (CagA) *H. pylori* strain [178]. In another study, the risk ratios of GC was 3.1 (95%CI: 1.97–4.95) between *H. pylori* infected and non-infected persons [179]. The risk ratios, based on case–control study designs, varied between 1.6 and 7.9 in three published papers from two extensive prospective nutritional intervention trials of over 29,000 males at age of 50–69 years in Linxian, China and Finland [180–182].

Our estimate of the prevalence of GC due to *H. pylori* infection in cross sectional studies was 19.46% (95% CI: 18.34—20.57) [*N* = 69; I^2^ = 98.59%], Therefore, from every 1000 H. pylori positive patients, 183 to 206 individuals get GC.

The simple infection markedly increases the cancer risk when compared to a healthy stomach. The risk varies between the populations with the highest and lowest by 15 to 20 times. East Asia (China and Japan), South America, Eastern Europe, and Central America are the high-risk regions. North and East Africa, North America, Southern Asia, New Zealand, and Australia are the low-risk regions [183].

Our study noted the lowest prevalence of GC in *H. pylori* positive patients from the Sweden (Prevalence: 0.07%; 95% CI: 0.06–0.09) [107] and the highest from the Japan (Prevalence: 90.90%:95% CI: 83.61–95.14) [161].

This difference may be due to the following reasons: dietary habits, socio-economic status and racial disparities. Suerbaum et al. [184] have mentioned that populations with lower socioeconomic status were more likely to be infected with *H. pylori*. Data based on National Health and Nutrition Examination Surveys of the United States have also shown that racial disparities played a certain role in the prevalence of *H. pylori*. The prevalence of *H. pylori* in African Americans was higher than whites [185]. The findings of the studies showed that Blacks and Hispanics consistently have higher *H. pylori* prevalence, serologic markers, and histologic signs than whites. Generally, the prevalence of CagA in adult people with *H. pylori* positivity ranged from 71%-90% in blacks, 64%-74% in Hispanics, and 36% to 77% in whites. Studies that amplified the VacA m allelic region for genomic characterization discovered that Blacks and Hispanics were more likely than whites to carry the virulent VacA-m1 genotype [186]. It has been hypothesized that racial discrepancies associated with *H. pylori* are contributed to GC incidence and mortality.

The evidence that is currently available implies that practitioners should be aware that the prevalence of *H. pylori* varies depending on race [187]. Perhaps it would be better if we personalized GC prevention and improved clinical management for all patients.

The results of subgroup analysis, based on our design, the highest and lowest prevalence was observed in prospective case series (pooled prevalence: 23.13%; 95% CI: 20.41 − 25.85; I^2^: 97.70%) and retrospective cohort (pooled prevalence: 1.17%; 95% CI: 0.55 − 1.78; I ^2^: 0.10%). The highest and lowest prevalence of GC in *H. pylori* patients was observed in America (pooled prevalence: 18.06%; 95% CI: 16.48 − 19.63; I^2^: 98.84%) and Africa (pooled prevalence: 9.52%; 95% CI: 5.92 − 13.12; I^2^: 88.39%) continents, respectively.

Steady declines in GC incidence rates have been observed worldwide in the last few decades [183]. The general declining incidence of GC may be explained by higher standards of hygiene, improved food conservation, a high intake of fresh fruits and vegetables, and by *H. pylori* eradication [188]. Current treatment for *H. pylori* infection includes antisecretory agents or bismuth citrate plus two or more antimicrobials. Clarithromycin and metronidazole are the most commonly used antibiotics to treat *H. pylori* infection. Increasing resistance of *H. pylori* to metronidazole and clarithromycin has made current therapies with these antibiotics less successful [68]. Bismuth triple therapy is not very effective in the presence of a high prevalence of metronidazole resistance, unless higher doses of metronidazole are prescribed to increase the cure rate of therapy. Resistance to the major anti-*H pylori* antibiotics, the final duration of therapy, and the prescribed antibiotic dose are all factors that affect the efficacy of therapy. Host genetic polymorphisms may also influence the efficacy of therapy [189].

The results of our study indicated a significant heterogeneity (*p* < 0.001) in the prevalence of *H. pylori* in GC across different geographical regions. The result of univariate meta‐regression analysis showed the age, sample size and study design possess significant effect on the studies heterogeneity and have eligible to include to multiple model. The results of multiple meta‐regression analysis showed the just age have a significant effect on the studies heterogeneity. The R^2^-adj for multiple model was 13.63% and this mean the age, sample size and study design explained the about 14% of total heterogeneity of prevalence. This was in accordance with a recent study that assessed the prevalence of *H. pylori* in gastrointestinal disease cases [97]. Study performed by Spineli et al. [98] revealed that subgroup analysis may not be powerful enough to test for relationships between variables when fewer studies are involved. However, type of sample was significantly associated with *H. pylori* prevalence [184]. Although subgroup analysis and meta-regression were performed to minimize the heterogeneity across the included studies, significant heterogeneity still could be observed in subgroup analysis. Moreover, some important factors like drinking and dietary habit could not be extracted from the included studies, which might have potential influence on the heterogeneity.

Therefore, these results should be considered with caution and more studies are needed to further confirm these results in the future.

In general, limitations of meta-analyses are that the validity is dependent on the quality of the included studies, on heterogeneity between studies, and on possible publication bias; but we tried to deal of them by statistical manner. Indeed we dealt to heterogeneity by using random effects model, subgroup and meta-regression analysis. Also we tried to deal publication bias by use the fill and trim method to estimate the publication-bias-adjusted-pooled.

## Conclusions

In our study by evaluate the 149 studies and 352,872 sample size illustrated that prevalence of GC in patient with *H. pylori* was considerable. But the rate was varied based on different subgroups so that the rate was highest among in America continent but was lowest in Africa continent. Also, using meta-regression and assessment the effect of several variables, indicated that age, sample size and study design explained the about 14% of total heterogeneity. It is advised to launch appropriate control guidelines for high-risk region. The risk of different factors should also be taken into account when developing GC decrease strategies, even though *H. pylori* eradication may be a promising method for preventing the disease.

## Data Availability

All data generated or analyzed during this study are included here and are available from the corresponding author on reasonable request.

## Abbreviations

GC: Gastric cancer
NOS: Newcastle Ottawa scale
PRISMA: Preferred Reporting Items for Systematic Reviews and Meta-Analyses
RR: Risk ratio
